# Analysis of modularity and integration suggests evolution of dragonfly wing venation mainly in response to functional demands

**DOI:** 10.1098/rsif.2018.0277

**Published:** 2018-08-29

**Authors:** Alexander Blanke

**Affiliations:** Institute of Zoology, Biocenter Cologne, University of Cologne, Zülpicher Str. 47b, 50674 Köln, Germany

**Keywords:** insect, wings, geometric morphometrics, modularity, integration, allometry

## Abstract

Insect wings show a high variability in wing venation. Selection for function, developmental pathways and phylogeny likely influenced wing vein diversification, however, quantitative data to estimate these influences and their interplay are missing. Here, it is tested how dragonfly wing vein configuration is influenced by functional demands, development, phylogeny and allometry using the concepts of modularity and integration. In an evolutionary context, modules are sets of characters that evolve in relative independence to other characters, while integration refers to a high degree of association between subparts of a structure. Results show allometric and phylogenetic signal in the wing shape variation, however, patterns of integration and modularity are not influenced by these two factors. Overall, dragonfly wings are highly integrated structures with almost no modular signal. Configuration changes in one wing vein or wing area thus influence wing shape as a whole. Moreover, the fore- and hindwings correlate with each other in their evolutionary shape variation supporting biomechanical data of wing interdependence. Despite the overall high degree of evolutionary integration, functional hypotheses of modularity could be confirmed for two wing areas, the arculus–triangle complex at the base of the wing which is responsible for passive wing folding especially during flapping flight and the location of the pterostigma, a coloured wing cell which is more heavy that other wing cells and passively regulates wing pitch as well as critical flight speeds during gliding. Although evolving as distinct modules, these specific vein regions also show high integration and evolve at the same rates like the whole wing which suggests an influence of these structures on the shape evolution of the rest of the wing. Their biomechanical role as passive regulators of wing corrugation and wing pitch suggests that these structures decisively influenced the evolution of advanced modern flight styles and explains their retention once they had evolved early within the lineage Odonatoptera.

## 1. Introduction

Modularity and integration are central concepts to understand how the organization of morphological structures influences the evolution of the phenotype across taxa [35]. In an evolutionary context, modules are sets of characters that evolve in relative independence to other characters, while integration refers to a high degree of association between subparts of a structure [26,29,35]. All parts of an organism evolve integrated to a certain degree. Consequently, the homogeneity of the integration signal across a composite structure as opposed to its potential modular organization, and the underlying causes are of concern to understand the evolution of a particular phenotype. For example, allometry, the influence of size on shape, can alter levels of integration [26,27,33] while selection for a specific function can result in evolutionary modularity [28]. Therefore, if modularity is assessed, it also has to be determined to what extent allometry supports evolutionary integration and how this impacts the modular signal.

Insect wings have been studied on an intraspecific level to decipher the causes and relative strengths of integration and modularity signals [13,30–32]. Overall, it was found that integration across the whole wing is high and that developmental compartmentalization [32] or functional subparts [31] do not translate into a modular shape variation of certain wing areas (but see Munoz-Munoz *et al*. [36]). Wings seem to vary in shape as one integrated unit. In an evolutionary framework, patterns of integration and modularity have so far not been investigated for insect wings. Here, dragonfly wing venation is used to investigate such patterns and to test commonly suggested functional and developmental influences [8,14,16–18] on dragonfly wing evolution. The extensive wing venation system in dragonflies compared to the more derived and largely reduced wing venation found in dipteran taxa such as *Drosophila* theoretically should enable the study of potential modular shape variations on a much higher level of detail, i.e. for single wing veins and their potential covariation with other parts of the wing.

**Figure 1.**
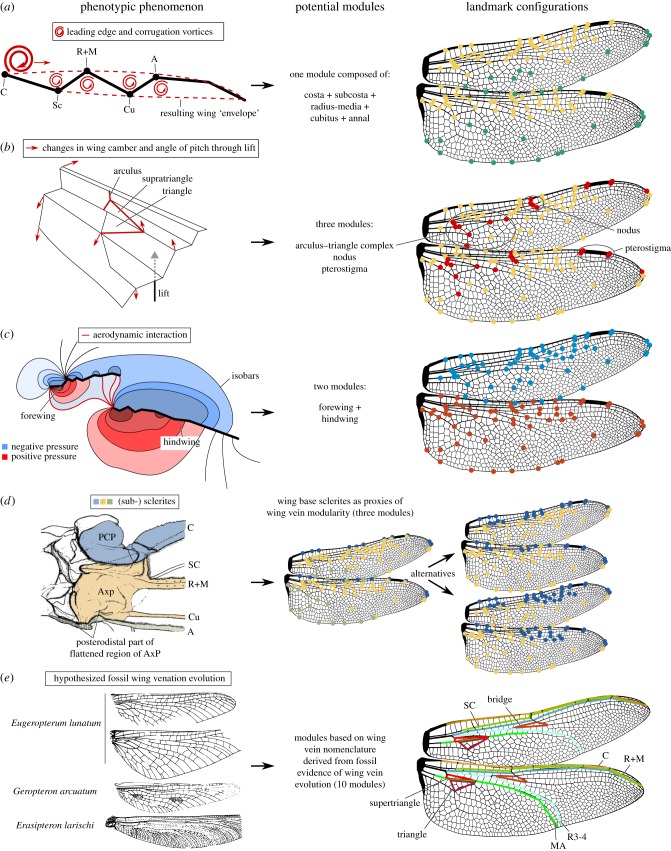
Illustrations of the hypotheses concerning potential functional, developmental, and phylogenetic influences on wing vein shape variation. (*a*) Overview of the vortex structure occurring during flight in dragonflies. A leading edge vortex travels over the wing during each stroke and smaller vortices in the valleys between the veins help to form a smooth envelope increasing lift capability. Modified after Bomphrey *et al*. [12]. (*b*) Effect of an upward directed force applied in the distal median area of the wing (black and grey arrow) on the wing vein region around the arculus–triangle complex. The trailing edge is lowered which improves camber and attitude in response to this loading. Modified after Wootton *et al*. [50]. (*c*) Fore- and hindwing pressure isobars illustrate how positive and negative pressure regions of each wing connect with each other (here at 25% of the wing length in *Aeshna grandis*). Modified after Bomphrey *et al*. [12]. (*d*) Wing vein insertions at sclerites of the wing base in Odonata, here shown for *Tanypteryx pryeri* after Ninomya & Yoshizawa [38]. (*e*) Wing vein modules based on the nomenclature of Riek & Kukalová-Peck [43] which is based on the wing venation patterns found in fossil Odonatoidea and postulated wing vein evolution. Modifier after Riek & Kukalová-Peck [43]. Abbreviations: C, costa (leading edge of the wing until the nodus); Sc, subcosta (second longitudinal vein, leading edge distal of nodus); R+M, radius and media veins; Cu, cubitus (fifth longitudinal vein); A, anal vein. The locations of these veins and other vein structures are indicated in *a*, *b* and *e*.

For dragonfly wings the configuration of the costal region (the leading edge of the wing) is believed to have an influence on the configuration of more posteriorly located wing parts [52] (figure 1*a*). This could be related to the occurrence of vortices, small circular airflows which are essential to generate favourable lift-to-drag ratios during flight [19,22]. Like in most insects, dragonfly wings generate leading edge vortices at the anterior part of the wing [12]. The corrugation of the dragonfly wing at its frontal part (mainly the costa–subcosta–radius veins) furthermore generates smaller vortices between the veins which were reported to form a ‘smooth envelope’ of the wing profile enhancing lift [12,20,37] (figure 1*a*). Regarding the variation in wing vein configuration one would expect that especially the leading edge veins are subject to modularity with respect to the rest of the wing, while there should be a strong integration signal among these three veins in order to maintain favourable vortices for each species.

Three other characteristic areas, the triangle, the nodus and the pterostigma (figure 1), are believed to be major developments during dragonfly wing evolution, which made the modern flight styles of Odonata possible and largely influenced the configuration of other wing parts [39,50] (figure 1*b*). Functional dependency was even suggested to occur between forewings (FWs) and hindwings (HWs) since the FW can influence the airflow around the HW [12,48] (figure 1*c*). However, it has so far not been tested whether dragonfly FWs and HWs show correlated shape variation across species or whether certain wing areas such as the triangle and the nodus show distinct shape variations with respect to the shape variation of the rest of the wing, i.e. whether they are distinct modules or highly integrated, or both.

Dragonfly wing shape and compartmentalization could have also been influenced by developmental pathways. The imaginal discs of fly wings such as those of *Drosophila* are developmentally subdivided into anterior, posterior, dorsal and ventral compartments during development [10]. Although there is currently no information available whether dragonfly wings are similarly compartmentalized during development, the dragonfly wing base is composed of two peculiar sclerites, the proximal costal plate (PCP) and the axillary plate (AxP) [38] (figure 1*d*) which are probably a fusion product of more sclerites present in the groundplan of winged insects. If sclerite configuration is a proxy for wing vein development, the vein groups attached to the sclerites should show modularity with respect to each other.

Furthermore, the nomenclature of dragonfly wing veins and their homology is based on fossil evidence about fusion, reduction and modification of branching patterns of certain wing veins e.g. the radius and the media [43]. Although this wing venation system was a matter of debate [9,15], it is presently considered most correct [42]. If the configuration of wing veins is influenced by their respective ancestral conditions, each of these veins might show modular shape variation with respect to the rest of the wing. Here, a quantitative assessment of modularity and integration of dragonfly wing venation is presented based on the above-mentioned functional and developmental hypotheses using a geometric morphometric approach.

## 2. Material and methods

The dataset comprises 189 species of dragonflies from all currently recognized families (species list in electronic supplementary material, table S1). Wings were removed from specimens and placed under a square glass plate on an Epson Perfection 4870 PHOTO scanner, scanning was performed at a resolution of 1200 DPI in colour. Scans were then edited to remove debris and minor defects and finally converted to 1200 DPI bitmap images.

### 2.1. Landmark definitions

A series of 121 homologous landmarks, 60 in the FW and 61 in the HW were chosen to represent overall wing shape and major wing veins (figure 1). The description of landmark points follows the wing vein nomenclature of Riek & Kukalová-Peck [43]. As not all landmarks were present in all specimens, the number of species and the landmark set were reduced to maximize species and landmarks at the same time. The final dataset contained 174 species and 59 landmarks in the FW and 55 landmarks in the HW (electronic supplementary material, table S2).

### 2.2. Geometric morphometrics

Landmarks were placed in Blender v. 2.77 (www.blender.org) and exported into the R software environment. A Procrustes superimposition was carried out to correct for effects of rotation, translation and size [23,45] using geomorph v. 3.0.5 [5]. Principal component analysis was performed to investigate the variance associated with the shape variables.

The phylogeny presented by Letsch *et al*. [34] was used to test for the phylogenetic signal (i.e. the tendency for closely related species to display similar trait values due to their common ancestry) in the shape data [1,11] whereas the centroid size of the Procrustes aligned shape data was used to test for allometric signal. As both phylogenetic and allometric signals were detected, all analyses were performed on the uncorrected data and on the residuals of a phylogenetic generalized least-squares (PGLS) regression of shape against centroid size.

### 2.3. Wing mechanics and coloration

To test whether energetic and aerodynamic requirements correlate with wing shape variation, the non-dimensional radius of the second moment of wing area r_2_(S) was calculated for the FW and HW of each species [21]. This parameter is proportional to the square root of the aerodynamic forces in hovering and flapping flight and thus also relates to the energy requirements for flight and the agility of species [21,46,49]. Lower values indicate a more proximal distribution of the wing area which also indicates a wider range of executable flight speeds [21,51]. Five hundred chordwise wing spans per wing were extracted using custom scripting in ImageJ v. 1.51s which enabled calculation of the r_2_(S) after Ellington [21].

Furthermore, correlation of, and interaction between, wing pigmentation patterns, habitat and the r_2_(S) with wing shape was tested. Many dragonflies use their wing pigmentation as secondary sexual traits and it was shown that such pigmentation can influence wing shape [40]. Hence, pigmentation groups were defined according to Outomuro [40] (electronic supplementary material, table S1) and subsequently tested for correlation of wing pigmentation. Letsch *et al*. [34] showed that speciation is correlated with habitat. It was therefore also tested whether wing shape variation is correlated with the colonization of lentic or lotic habitats (electronic supplementary material, table S1).

### 2.4. Modularity and integration

Five hypotheses of a potential modular evolution of wing parts within a wing or the whole wing were tested for modularity and integration. *Hypothesis A* (figure 1*a*) is based on the occurrence of vortices especially at the leading edge of the wing and within the corrugated areas at the anterior part of the wing [12,20,37]. It is hypothesized that wing regions with vortices show a modular shape variation with respect to the rest of the wing. Accordingly, the costa, subcostal, radius, and triangle regions were defined as one module with respect to the rest of the wing. *Hypothesis B* (figure 1*b*) is based on the functional morphology of the triangle–arculus complex, the nodus area and the location of the pterostigma. Dragonfly flapping flight results in passive changes of wing camber and angle of pitch through lift. Wooton *et al.* [50] found that an uplift force in the triangle area leads to widespread configuration changes along the wing during the downstroke by cambering and increasing its angle of pitch. The nodus, with its peculiar joint design [47], was suggested as an evolutionary step towards the agile flight styles of modern Odonata [14,50,52]. Finally, the location of the pterostigma was suggested as an inertial regulator of wing pitch thus leading to increased speed limits during flapping and gliding flight [39]. Accordingly, it is hypothesized that the triangle–arculus complex, the nodus area and the pterostigma each show a modular shape variation with respect to the rest of the wing. For each of the three ‘sub-hypotheses’ the respective landmark set was subsetted and tested against the rest of the wing landmark set. For *hypothesis C* (figure 1*c*) it was tested whether the FWs and HWs correlate in shape variation. Since the wings are not physically connected to each other, independent Procrustes superimpositions on each of the two landmark sets followed by a two block partial least-squares analysis [44] were carried out in geomorph v. 3.0.5 [5]. *Hypothesis D* (figure 1*d*) is based on the configuration of wing base sclerites in dragonflies. The costa is attached to the proximal plate (via a distal plate), radius, media, subcosta and cubitus veins arise from the anterior margin of the AxP and the anal vein from a posterodistal part of a so-called ‘flattened region’ of the AxP [38]. Accordingly, three modules encompassing the landmarks of the costa vein (module1), radius, media, subcosta and cubitus veins (module2) and A vein (module 3) were defined. Alternatives to this three module scenario with only two modules (based on the two sclerites PCP and AxP) were also tested. For this, alternative designations of the more distal wing parts we also taken into account (figure 1*d*, right). Finally, *hypothesis E* (figure 1*e*) is based on the wing vein nomenclature, i.e. each main wing vein was assumed to be an independent module compared to the rest of the wing. The wing vein nomenclature in dragonflies is based on fossil evidence and relates to historical patterns of wing vein fusion and branching [43]. All tests for modularity and integration (except hypothesis C) were carried out as implemented in geomorph v. 3.0.5 using the ‘phylo.integration’ and ‘phylo.modularity’ functions [2,5,4]. It is noted that their default is a Brownian motion model of evolution and that model misspecifications when using other models for the analysis of high-dimensional multivariate data are currently discussed [3]. Allometry influences patterns of evolutionary integration and can therefore also affect the detection of modularity [26,27,33]. To investigate the influence of allometry the integration and modularity tests were carried out on the original data as well as on the residuals of a PGLS regression of shape against centroid size. If a module in a part of the wing was found, it was tested whether the tempo of morphological evolution of that module was different to the rest of the wing using the evolutionary rate parameter under a Brownian motion model of evolution as implemented in geomorph v. 3.0.5. The evolutionary rate parameter quantifies whether the shape variation in a landmark group varies significantly to the rate in another group given the phylogenetic history of the species. In the following, the first coefficient of determination (*R*^2^) is the FW value whereas the second one is the HW value.

## 3. Results

**Figure 2.**
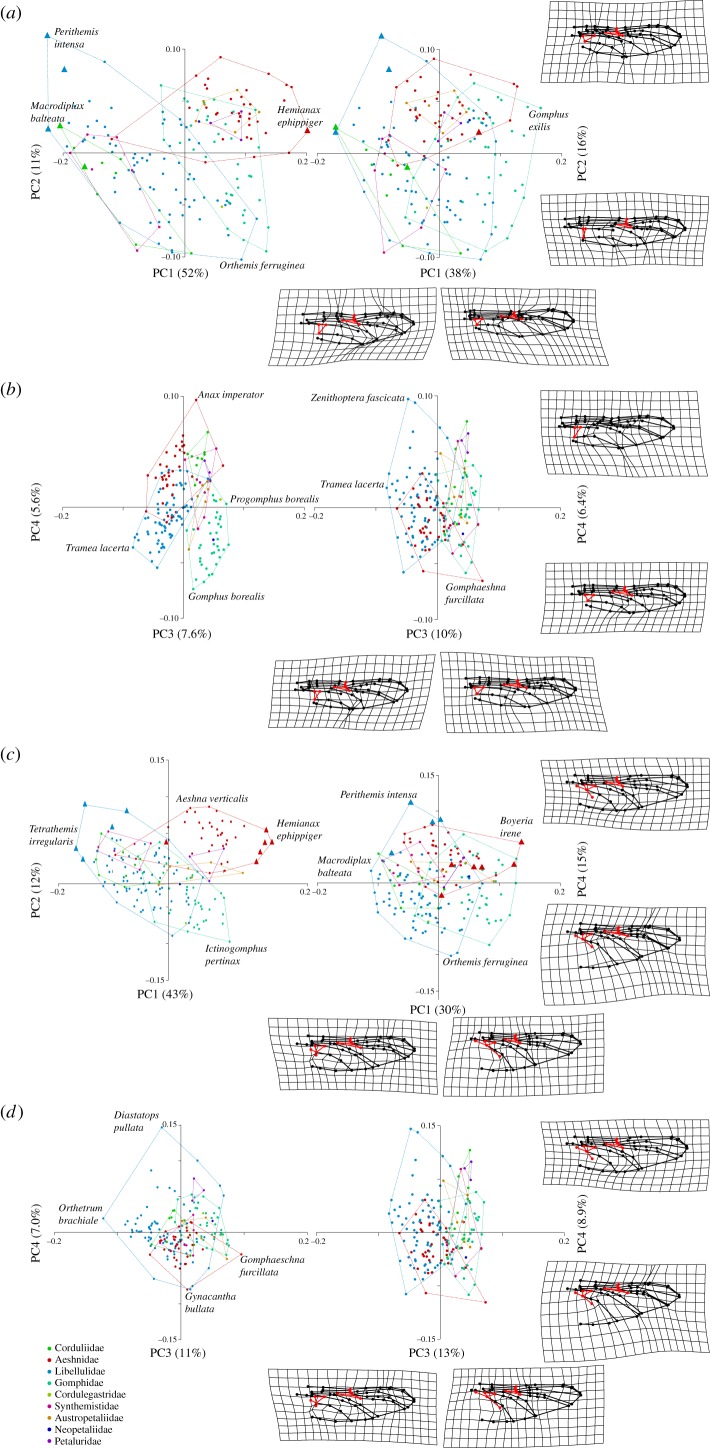
Plots of the uncorrected (left) and the phylogeny and allometry corrected (right) first four principal components for the fore- and hindwings of all studied species. (*a*) First two PCs of the forewing. (*b*) PCs 3 and 4 of the forewing. (*c*) First two PCs of the hindwing. (*d*) PCs 3 and 4 of the hindwing. Families are indicated with dashed envelopes. Outliers in shape variation are indicated with triangles, the ‘extreme’ species for each PC are named. Thin plate splines are only given for the PGLS data respectively. Triangle, nodus area and bridge landmarks are highlighted in red to facilitate recognition of shape changes.

A phylogenetic (*p* = 0.001) as well as allometric signal (*R*^2^ = 0.26/0.21; *p* = 0.0001) was detected in the FW and HW datasets. The Kmult value before allometric correction was 0.23 for both wings (after allometric correction 0.18). The first two PC axes accounted for 51% and 11% of the overall variability in the FW while they accounted for 43% and 12% in the HW (table 1). 3D plots of the first three PCs show that before allometric correction Gomphidae + Aeshnoidea + Cordulegastridae are well separated from Libelluloidea (electronic supplementary material, figure S1 and videos S1 and S3). A significant amount of shape variation within PC1 is attributable to allometry while all other PCs are little affected (table 1; figure 2*a* and *c*). After phylogenetic and allometric correction the first two PC axes accounted for 38% and 15% of the shape variability in the FW while they accounted for 30% and 15% in the HW (table 1). Three-dimensional plots show that the principal separation between Libelluloidea seems to collapse but the taxonomic separation of families nevertheless remains (*R*^2^ = 0.32/0.30; *p* = 0.001; table 2; electronic supplementary material, figure S1 and videos S2 and S4). Both, the uncorrected and the allometry and phylogeny free wing shapes of the FWs and HWs furthermore showed a significant correlation with the second moment of wing area (table 2). Most of the tested characteristics also showed very minor significant interactions between each other (*R*^2^ ∼ 0.002–0.04 depending on the interaction term; see table 2).

**Table 1.** Summary of the principal components of shape variation for the uncorrected and allometry and phylogeny corrected shape data.

	forewing				hindwing			
	uncorrected		allometry and phylogeny corrected		uncorrected		allometry and phylogeny corrected	
	s.d.	% of total variance	s.d.	% of total variance	s.d.	% of total variance	s.d.	% of total variance
PC1	0.0974	0.519	0.0712	0.376	0.0775	0.425	0.0574	0.299
PC2	0.0457	0.114	0.0457	0.155	0.0413	0.121	0.0409	0.152
PC3	0.0373	0.076	0.0370	0.102	0.0399	0.112	0.0375	0.128
PC4	0.0319	0.056	0.0294	0.064	0.0315	0.070	0.0314	0.089
PC5	0.0294	0.047	0.0287	0.061	0.0238	0.040	0.0236	0.050
PC6	0.0258	0.037	0.0247	0.045	0.0229	0.037	0.0206	0.038
PC7	0.0221	0.027	0.0207	0.032	0.0190	0.026	0.0186	0.031
PC8	0.0181	0.018	0.0176	0.023	0.0179	0.023	0.0174	0.027
PC9	0.0164	0.015	0.0163	0.020	0.0156	0.017	0.0156	0.022
PC10	0.0142	0.011	0.0142	0.015	0.0146	0.015	0.0145	0.019

**Table 2.** Correlations of shape variation of fore- and hindwings with taxonomic units (families), wing coloration and second moments of wing area r_2_(S) of fore- and hindwings together with their respective interaction terms. Values in italics are significant *p*-values. *R*^2^ = coefficient of determination; *p* = probability value.

	forewing		hindwing	
	*R*^2^	*p*	*R*^2^	*p*
family	0.3295	*0.001*	0.3094	*0.001*
colour	0.0955	*0.001*	0.1007	*0.001*
r_2_(S) forewing	0.0560	*0.001*	0.0571	*0.001*
r_2_(S) hindwing	0.0037	*0.041*	0.0053	*0.003*
family:colour	0.0373	*0.001*	0.0369	*0.001*
family:r2f	0.0194	*0.001*	0.0256	*0.001*
colour:r2f	0.0189	*0.001*	0.0268	*0.001*
family:r2h	0.0192	*0.001*	0.0210	*0.001*
colour:r2h	0.0170	*0.001*	0.0201	*0.001*
r2f:r2h	0.0031	*0.036*	0.0033	*0.012*
family:colour:r2f	0.0133	*0.002*	0.0148	*0.001*
family:colour:r2h	0.0072	*0.007*	0.0064	*0.001*
family:r2f:r2h	0.0278	*0.001*	0.0301	*0.001*
colour:r2f:r2h	0.0054	0.061	0.0087	*0.002*
family:colour:r2f:r2h	0.0024	0.069	0.0035	*0.007*

Thin plate splines of the upper and lower extremes of PC shapes for the FW indicate that within PC1 the triangle (and to a minor extend the supratriangle and bridge) configurations are subject to shape changes while along PC2 especially the costal and subcostal cross-veins proximal and distal of the nodus and, more generally, the chordwise width of the wing showed changes in shape (figure 2*a*). For the HW, PCs1+2 related mainly to differences in the configuration of the anal loop and the width of the wing (figure 2*c*), while there are only minor differences in the configurations of RP1+2 (first and second posterior radii) and IRP2 (second intercallar posterior radius). PCs3+4 also coded mainly for variation on the anal loop area and the RP and IRP configurations (figure 2*d*).

**Figure 3.**
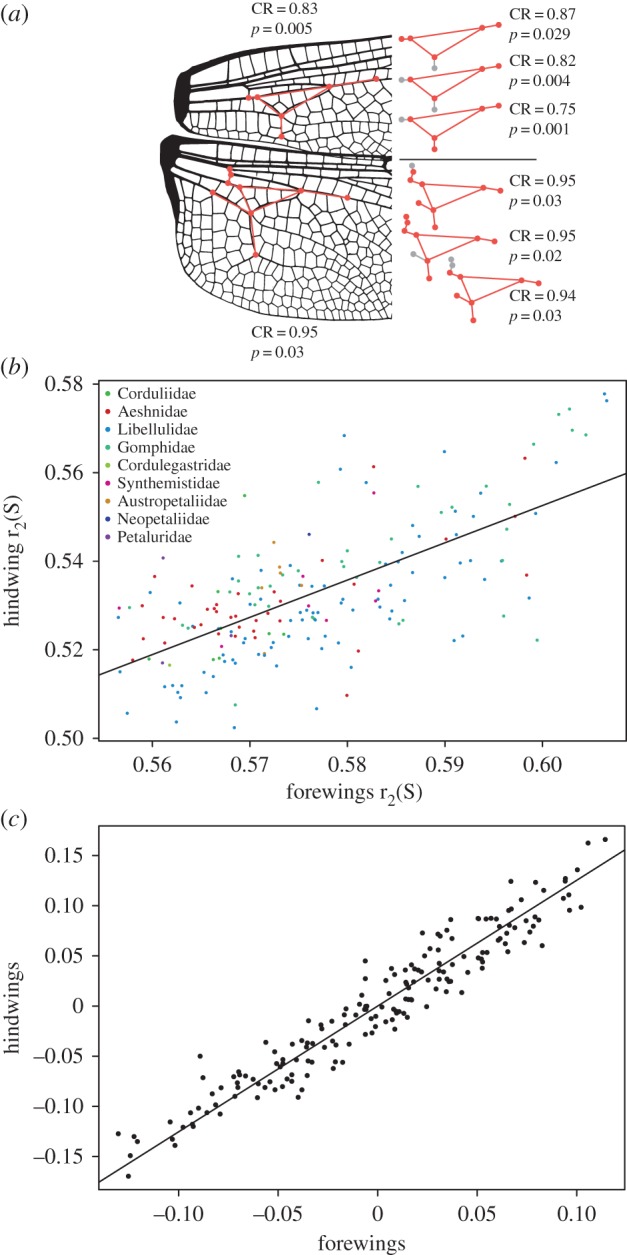
(*a*) Summary of the modular structure within the triangle–arculus complex and alternative modules tested. Parts missing in alternative configurations indicated in grey colour. The asterisk indicates significance (*p* < 0.05). (*b*) Regression of the r_2_(S) values of fore- and hindwings of all species (*R*^2^ = 0.46; *p* < 0.0001). Colour code for families as in figure 2. (*c*) Two-block partial least-squares analysis of the correlation in wing shape variation between fore- and hindwings (r-PLS = 0.96; *p* = 0.0001, see also figure 1*c*).

For each of the hypotheses of integration and modularity there was a strong integration signal (two-block partial least-squares (r-PLS) values: 0.79–0.97; *p* = 0.001; table 3; figure 3*a*) while a modular signal was found only for the passive wing folding hypothesis, i.e. the triangle–arculus complex in both wings (covariance ratio (CR) = ∼0.82–0.95; *p* = 0.004–0.03; table 3), the triangle within the uncorrected FW shapes (CR = 0.89; *p* = 0.02), and the pterostigma in the HW (CR = 0.93; *p* = 0.03–0.046). Both, the triangle–arculus complex and the pterostigma, showed non-significant differences in evolutionary rates compared to the rest of the wing for the corrected as well as the uncorrected wing shape data (triangle–arculus FW rate ratio = 1.26; *p* = 1/HW rate ratio = 2.57; *p* = 1; pterostigma FW rate ratio = 1.24; *p* = 0.97/HW rate ratio = 1.27; *p* = 1). Two-block partial least-squares analysis showed that the FWs and HWs strongly correlated in their shape variation (r-PLS = 0.96; *p* = 0.0001; figure 3*c*) and moderately correlated with r_2_(S) (r_2_(S) FW versus HW shape: r-PLS = 0.47; *p* = 0.0001/r_2_(S)HW versus FW shape: r-PLS = 0.58; *p* = 0.0001). The r_2_(S) values of FW and HW also moderately correlated with each other (*R*^2^ = 0.46; *p* < 0.0001) (table 4).

**Table 3.** Integration and modularity in the dragonfly *forewing* for each of the hypotheses mentioned in the text. Values in italics are significant *p*-values.

modules	integration				modularity			
	uncorrected		allometry and phylogeny corrected		uncorrected		allometry and phylogeny corrected	
	r-PLS	*p*	r-PLS	*p*	CR	*p*	CR	*p*
Wing venation [43]; hypothesis E (see also figure 1*e*)								
costa (C)	0.97	*0.001*	0.97	*0.001*	1.15	1	1.15	1
subcosta (Sc)	0.85	*0.001*	0.81	*0.001*	1.26	1	1.27	1
radius+media (R+M)	0.94	*0.001*	0.94	*0.001*	1.11	1	1.12	1
MA vein	0.83	*0.001*	0.83	*0.001*	1.08	0.98	1.10	1
triangle	0.87	*0.001*	0.87	*0.001*	0.89	*0.02*	0.92	0.10
supratriangle	0.89	*0.001*	0.89	*0.001*	1.17	0.86	1.16	0.96
bridge triangle	0.80	*0.001*	0.80	*0.001*	1.50	0.97	1.12	1
all triangles	0.96	*0.001*	0.96	*0.001*	1.09	1	1.64	1
R3-4	0.92	*0.001*	0.92	*0.001*	1.15	1.00	1.15	1.00
pterostigma	0.92	*0.001*	0.88	*0.001*	1.25	1	1.24	0.97
sclerites configuration [38]; hypothesis D (see also figure 1*d*)								
PCP sclerite	0.97	*0.001*	0.96	*0.001*	1.15	1	1.15	1
AxP sclerite	0.96	*0.001*	0.95	*0.001*	1.02	0.67	1.02	0.71
post. part of AxP	0.91	*0.001*	0.91	*0.001*	1.09	0.99	1.08	0.96
vortex occurrence and alternative sclerite configurations [12,38]; hypothesis A+D (figure 1*a*,*d*)								
Alt_1 p	0.97	*0.001*	0.95	*0.001*	1.16	1	1.16	1
Alt_2 p	0.86	*0.001*	0.87	*0.001*	1.08	1	1.08	1
Alt_3 p	0.86	*0.001*	0.87	*0.001*	1.08	1	1.07	1
passive folding [50]; hypothesis B and alternatives (figure 3*b*)								
Tri-arc complex 1	0.92	*0.001*	0.89	*0.001*	0.85	*<0.01*	0.82	*<0.01*
Tri-arc complex 2	0.92	*0.001*	0.91	*0.001*	0.90	*0.02*	0.87	*0.03*
Tri-arc complex 3	0.92	*0.001*	0.91	*0.001*	0.81	*<0.01*	0.75	*<0.01*
Tri-arc complex 4	0.92	*0.001*	0.92	*0.001*	0.76	*<0.01*	0.83	*<0.01*

**Table 4.** Integration and modularity in the dragonfly *hindwing* for each of the hypotheses mentioned in the text. Values in italics are significant *p*-values.

modules	integration				modularity			
	uncorrected		allometry and phylogeny corrected		uncorrected		allometry and phylogeny corrected	
	r-PLS	*p*	r-PLS	*p*	CR	*p*	CR	*p*
Wing venation [43]; hypothesis E (see also figure 1e)								
costa	0.96	*0.001*	0.96	*0.001*	1.01	0.15	1.05	0.55
subcosta	0.96	*0.001*	0.96	*0.001*	1.10	1.00	1.11	1.00
radius+media	0.96	*0.001*	0.96	*0.001*	1.14	0.99	1.18	1.00
MA vein	0.86	*0.001*	0.86	*0.001*	1.03	0.35	1.03	0.58
triangle	0.83	*0.001*	0.83	*0.001*	0.88	0.07	0.86	0.07
supratriangle	0.86	*0.001*	0.86	*0.001*	1.21	0.94	1.21	0.92
bridge triangle	0.80	*0.001*	0.80	*0.001*	1.57	1.00	1.53	1.00
all triangles	0.96	*0.001*	0.96	*0.001*	1.09	0.99	1.09	0.98
R3-4	0.95	*0.001*	0.95	*0.001*	1.05	0.63	1.03	0.46
pterostigma	0.92	*0.001*	0.90	*0.001*	0.93	*0.03*	0.93	*0.046*
sclerites configuration [38]								
PCP sclerite	0.96	*0.001*	0.95	*0.001*	1.01	0.15	1.05	0.55
AxP sclerite	0.94	*0.001*	0.94	*0.001*	1.02	0.61	1.03	0.93
post. part of AxP	0.86	*0.001*	0.87	*0.001*	1.10	1.00	1.09	1.00
vortex occurrence and alternative sclerite configurations [12,38]								
Alt_1 p	0.94	*0.001*	0.93	*0.001*	1.07	1.00	1.07	1.00
Alt_2 p	0.93	*0.001*	0.93	*0.001*	1.01	0.50	1.00	0.32
Alt_3 p	0.92	*0.001*	0.92	*0.001*	1.04	0.97	1.04	0.91
passive folding [50]								
Tri-arc complex 1	0.93	*0.001*	0.92	*0.001*	0.96	0.06	0.95	*0.02*
Tri-arc complex 2	0.91	*0.001*	0.92	*0.001*	0.96	*0.03*	0.94	*0.03*
Tri-arc complex 3	0.90	*0.001*	0.92	*0.001*	0.96	*0.03*	0.95	*0.03*
Tri-arc complex 4	0.90	*0.001*	0.93	*0.001*	0.95	0.05	0.95	*0.03*

## 4. Discussion

Integration and modularity are not mutually exclusive as a complex of characters can evolve in a modular fashion but still influence the shape evolution of the rest of the structure. The wing shape variation analysed here partly suggests such a pattern of strongly integrated but distinct modules (table 3). One example is the modular signal found for the arculus–triangle complex. This structure at the basal portion of both, FWs and HWs, was found to passively change the shape of the leading edge and the corrugation of the wing during flight depending on the particular lift forces during each flight phase [50]. Results suggest that this wing module is maintained functional *even* if other wing parameters such as the configuration of the RP and IRP veins or the number of cross-veins changes drastically. The occurrence of this arculus–triangle complex early in the evolution of Odonatoptera [50] and its retention in all Odonata supports the idea that this structure probably had a decisive influence on the evolution of advanced flight styles within Odonatoptera. It remains to be tested whether e.g. the wing veins in the area where one would expect a ‘triangle-like’ structure in the giant fossil Protodonata are in fact a functional analogue to the arculus–triangle complex of Odonata and whether these changes are attributable to an allometric effect in these fossil taxa. For fossils to be included in such an analysis, homology hypotheses concerning these wing vein areas in recent compared to fossil taxa need to be reassessed in order to be able to assign landmarks. Furthermore, the arculus–triangle complex shows modularity and integration at the same time. This means that although distinct in its shape evolution, this structure still influences the shape of other wing parts which is in line with the result that the structure evolves at the same rates like the rest of the wing.

Another example for concomitant integration and modularity is the configuration of the pterostigma which functions as a regulator of wing pitch and allows 10–25% increased critical flight speeds [39]. This distinct wing module is also strongly integrated and evolves at the same rates like the rest of the wing. It appears that stabilizing selection for functional performance led to the evolution of an arculus–triangle complex and a pterostigma which both supported the advanced flight styles of modern Odonata with high speed and manoeuvrability.

Apart from these two cases, the rest of the tested wing areas evolve highly integrated so that selective regimes acting on one wing part induce concerted changes in the rest of wing. Single wing veins as well as wing compartments based on developmental data of other insects (including alternative configurations, see figure 1*d* and table 3) do not evolve as distinct modules, so that hypothesis D, wing shape variation according to wing base sclerite configuration, and hypothesis E, relative independence of single wing veins, have to be rejected. Although not directly comparable, this is in line with previous studies carried out at the population level in more derived taxa: *Drosophila* wings showed a high integration of developmental compartments suggesting an integration of developmental processes across these compartments [27,32]. On the functional level it was tested for cricket wings whether developmental and genetic systems evolve to match the functional modularity of single wing compartments that are used in sound production, however, strong integration and no modularity was found [31]. Although the present study assessed macroevolutionary integration, these former studies indicate that developmental pathways in dragonfly wings might be highly integrated across the whole wing similar to the conditions found for *Drosophila*.

2B-PLS analyses suggest that changes in the wing vein configuration of the FW have an influence on the vein configuration of the HW. Given the relationship of shape changes to the indirect mechanical determinant measured (r_2_(S)) and the overall strong integration of wing veins it appears likely that the functional requirements of flight not only constrain modular wing vein changes but also induce concerted shape changes in both wings when they occur. These findings are supported by biomechanical studies of dragonfly flight which revealed an aerodynamic interaction of FWs and HWs during various flight manoeuvres [12,48].

In contrast to earlier results which suggested a strong relationship of wing coloration with wing shape for certain groups of Odonata [40,41], the present study showed only weak effects. This might be related to the large taxonomic sampling: The present study included species that show strong courtship behaviour and pre-mating displays of wing coloration (e.g. *Perithemis*, *Diastatops*) as well as species where such behaviour has not been reported but who still show wing pigmentations (e.g. other Libellulidae). Since wing pigmentations might also play a role for recognition of conspecific males and territoriality, and these territorial fights were suggested as the most demanding flight situations [12], wing shape in this larger and phylogenetically more diverse sample might be influenced by aerodynamic performance optimizations to ensure a high flight performance rather than direct female selection for mates with favourable display structures.

Tests for phylogenetic signal in the shape data suggest that species resemble each other less than expected under a Brownian model of evolution i.e. wings are more diverse in shape than expected from the phylogeny. A potential reason for this could be the high dispersal capabilities of dragonflies which might foster morphological diversification [24,25,34] or, alternatively or concomitantly, a many to one mapping of different wing forms onto a range of similar functional performance space [6,7]. Allometry also had a significant effect on shape with smaller libelluloids such as *Perithemis* and *Macrodiplax* and the largest aeshnids such as *Gynacantha*, *Neuraeschna* and *Staurophlebia* identified as outliers of the shape variation (figure 2; electronic supplementary material, figure S2 and videos S1–S4). Studies that investigate wing shape variation under different dispersal models as well as studies about the functional performance space variations of differently sized wings seem to be warranted to further our understanding of the evolutionary dynamics of insect wing shape variation.

## Supplementary Material

Supporting figure S1

## Supplementary Material

Supporting figure S2

## Supplementary Material

Supporting Table S1

## Supplementary Material

Supporting Table S2

## Data accessibility

The raw datasets supporting this article have been uploaded as part of the electronic supplementary material.
