# Supplementary material for: Analysis of modularity and integration suggests evolution of dragonfly wing venation mainly in response to functional demands
Source: J R Soc Interface. 2018 Aug 29;15(145):20180277. doi: 10.1098/rsif.2018.0277 (PMC6127186; doi:10.1098/rsif.2018.0277)
Supplement: Supporting figure S1 [file rsif20180277supp1.pdf]

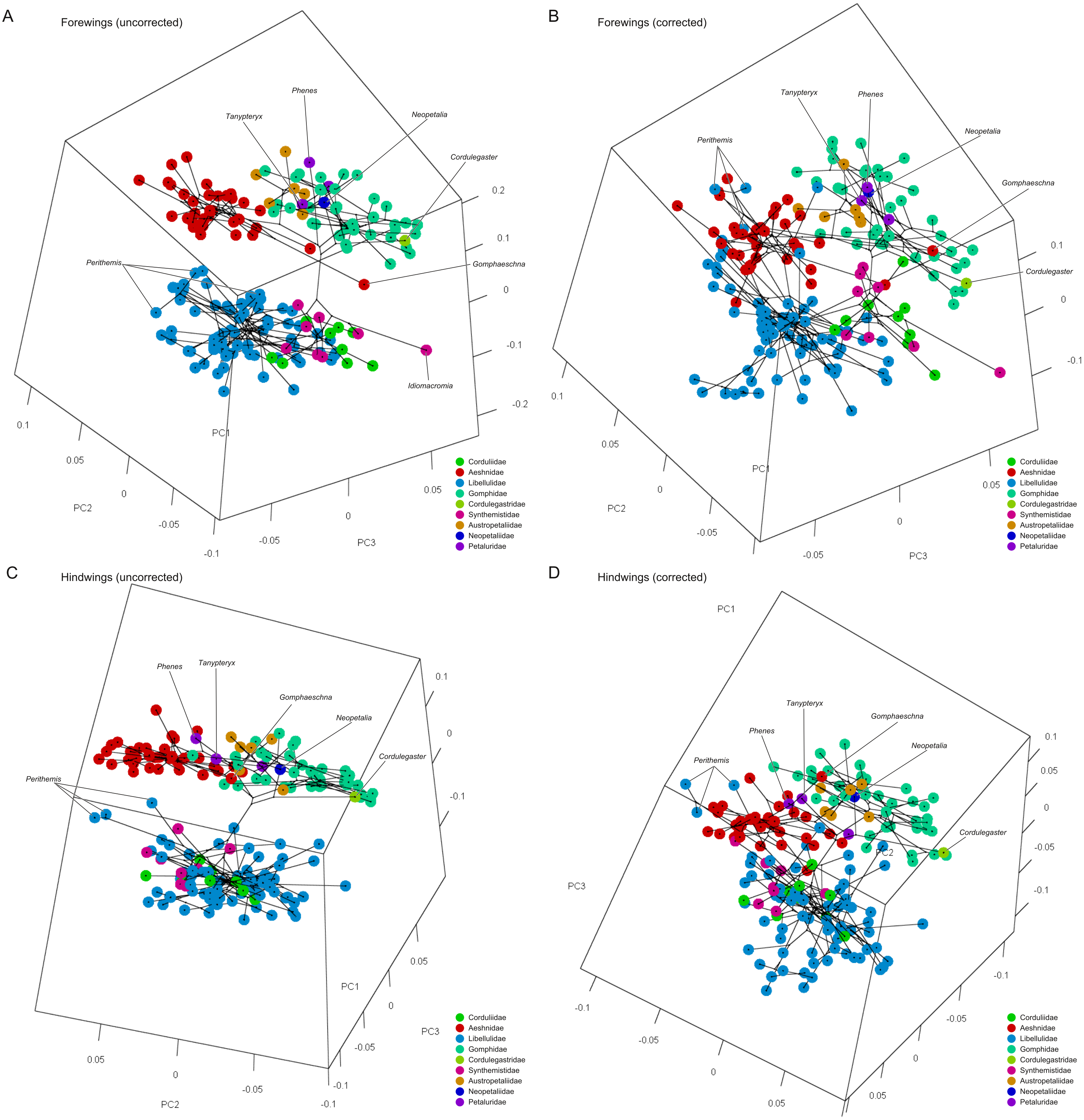

Supporting figure S1 Phylomorphospace plots of the first three principal components of the uncorrected (A+C) and allometry and phylogeny corrected (B+D) landmark datasets for the forewings (A+B) and hindwings (C+D). The branching pattern is based on the phylogeny of Letsch et al. (2016).

D
