# Supplementary figures and images for: Analysis of modularity and integration suggests evolution of dragonfly wing venation mainly in response to functional demands
Source: J R Soc Interface. 2018 Aug 29;15(145):20180277. doi: 10.1098/rsif.2018.0277 (PMC6127186; doi:10.1098/rsif.2018.0277)

All Specimens

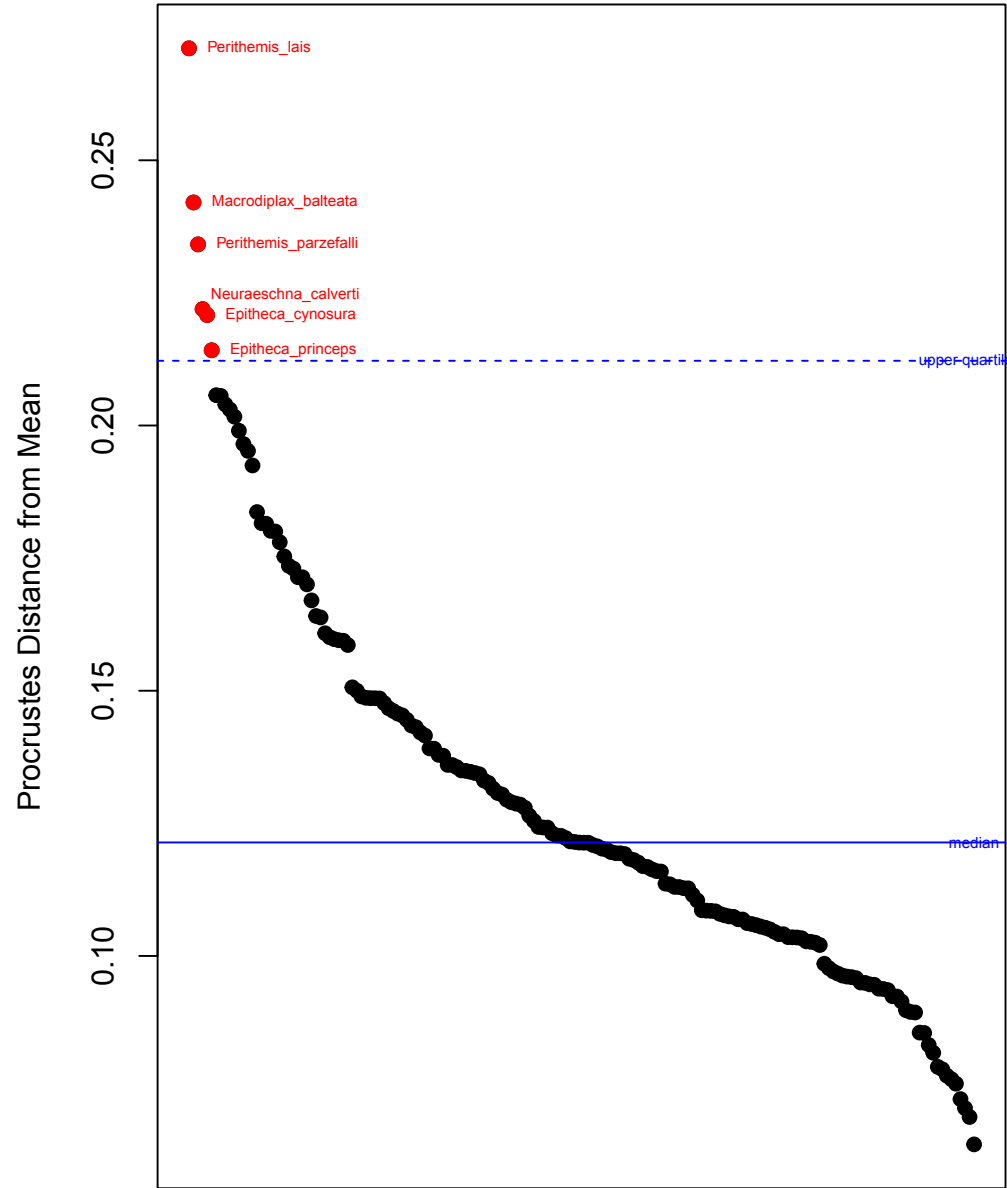

All Specimens

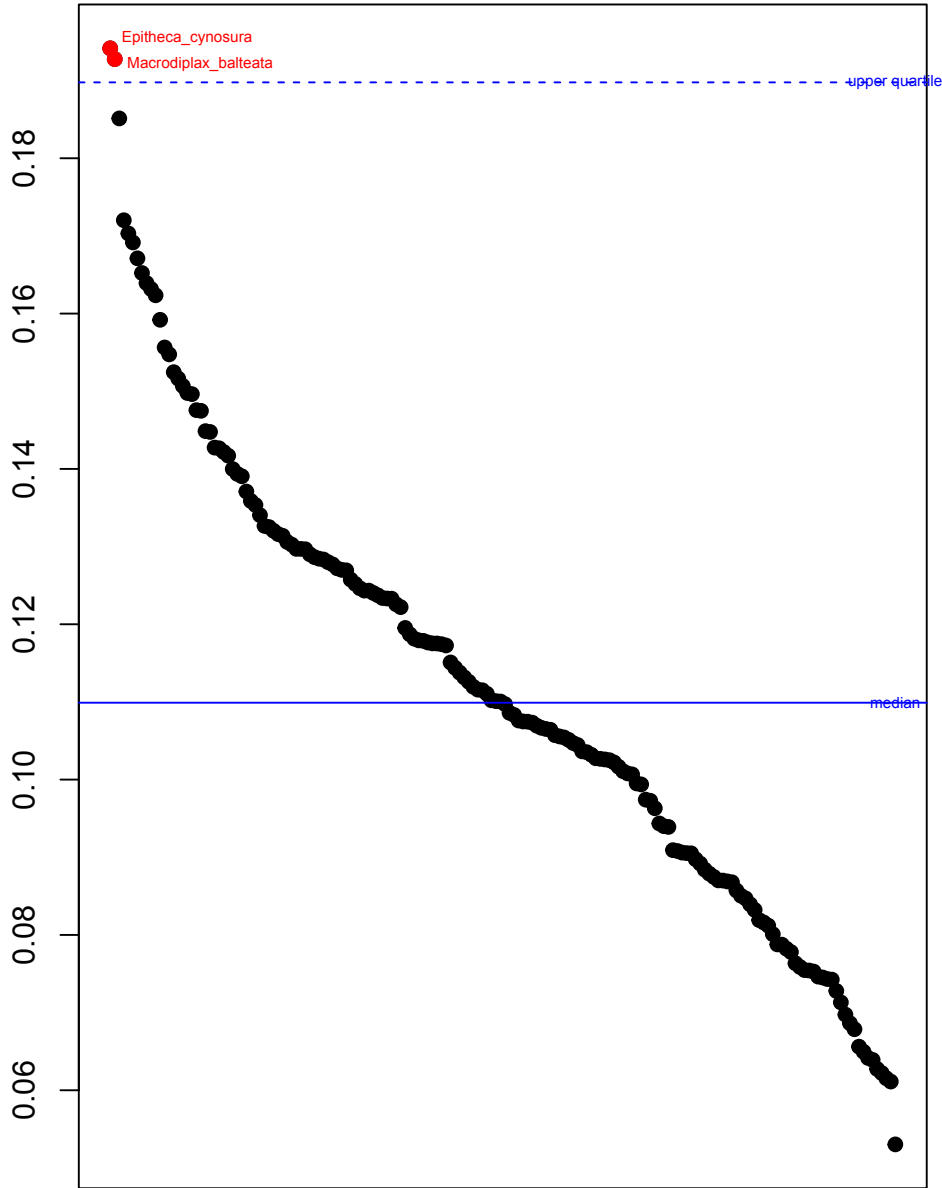

Procrustes Distance from Mean

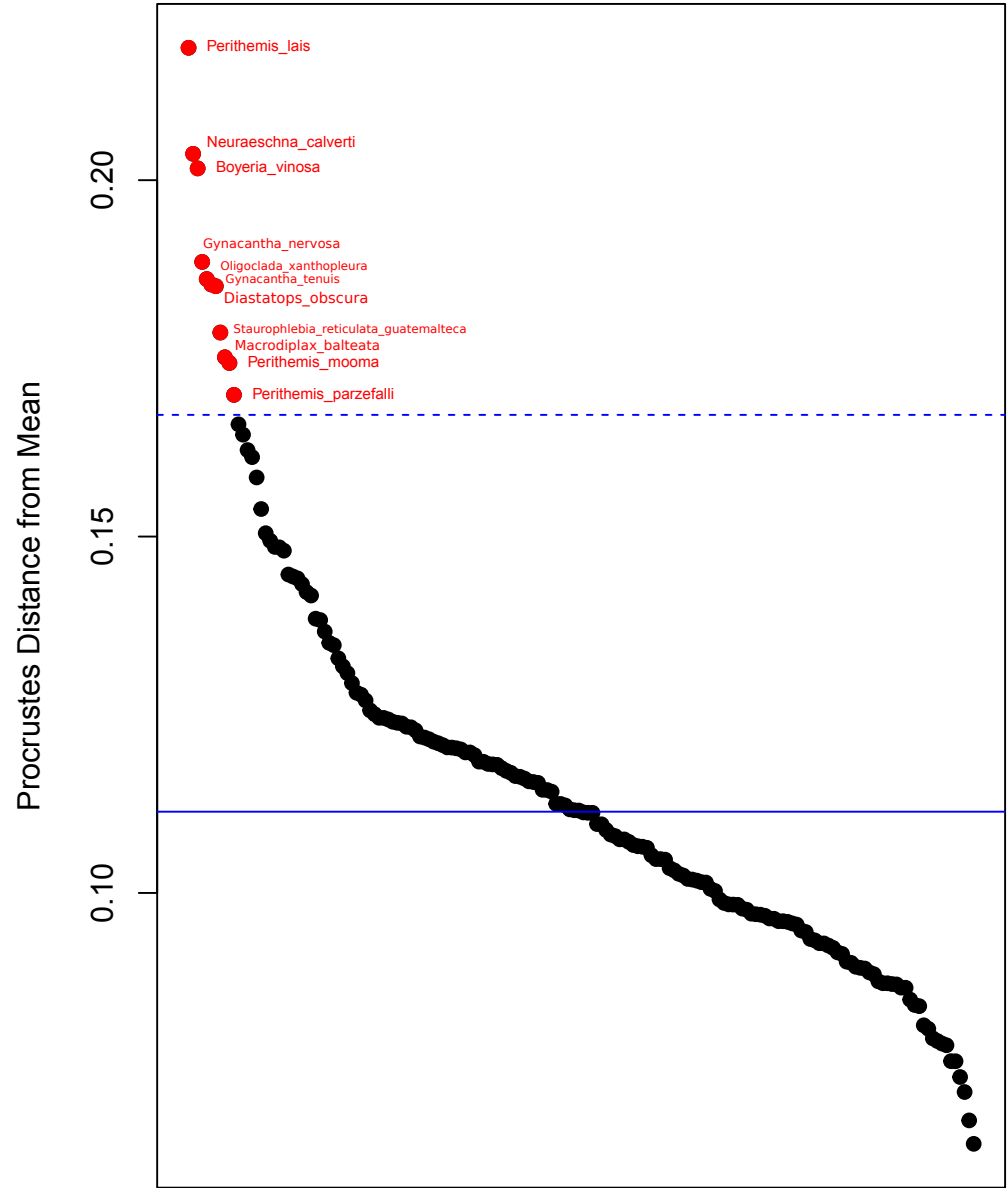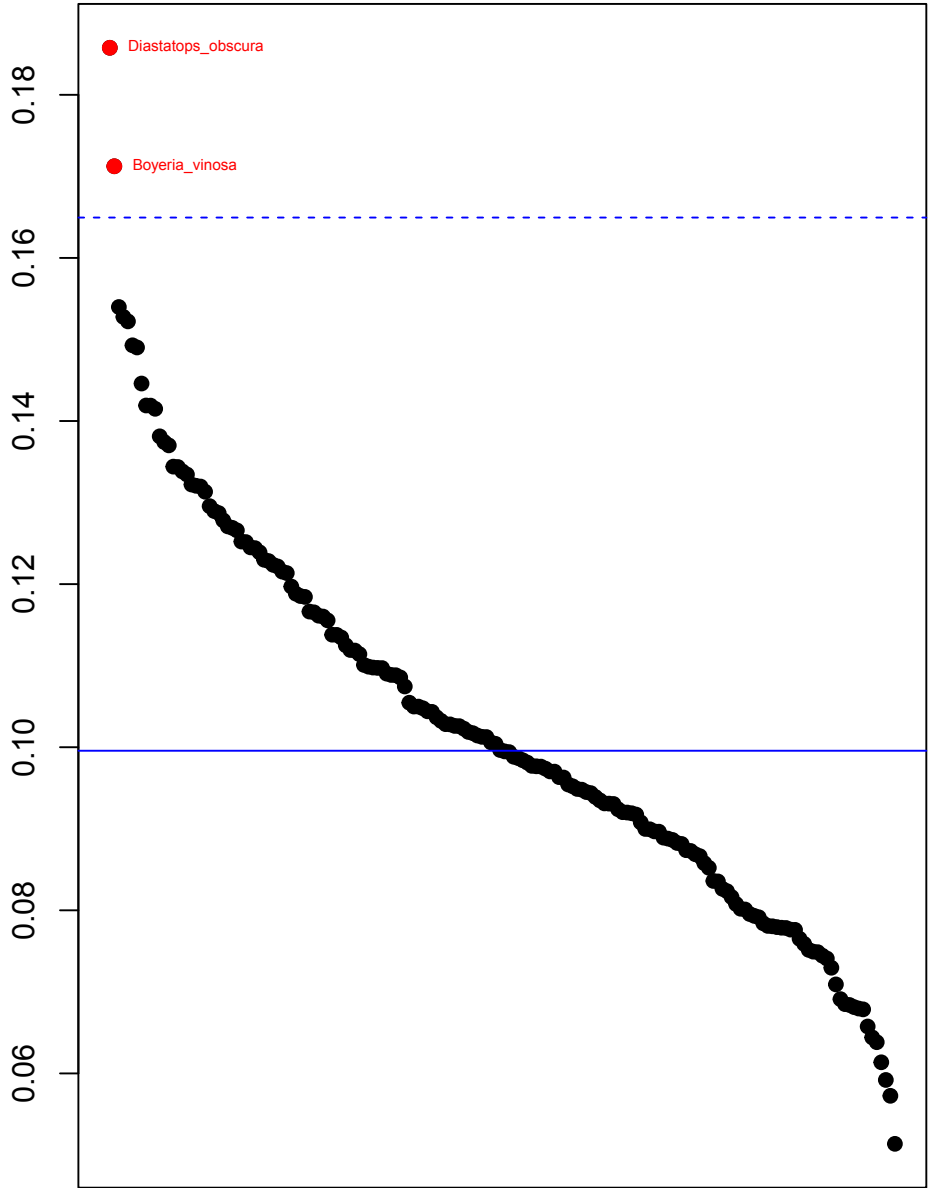

Supplement: Supporting figure S2 [file rsif20180277supp2.pdf]
